# Visual Attention to Emotional Faces in Children: An Eye-Tracking Study of Social Visual Attention

**DOI:** 10.3390/brainsci16070683

**Published:** 2026-06-29

**Authors:** Thaís de Fátima Bittencourt Oliveira, Erica de Freitas Marques, Guilherme Martins, Milena Fernandes de Oliveira, Leonardo Martins Guimaraes Rossi, Carlucio Gustavo Ribeiro Filho, Camila Fernanda Cunha Brandão, Lucas Rios Drummond, Lucas Túlio Lacerda, Michelle Morelo Pereira, Michael Jackson Oliveira de Andrade

**Affiliations:** 1 Laboratory of Neuroscience, Chronobiology, and Sleep Psychology, State University of Minas Gerais (UEMG), Divinópolis 35501-170, Brazil; thais.1665486@discente.uemg.br (T.d.F.B.O.); erica.1665488@discente.uemg.br (E.d.F.M.); guilherme.1656895@discente.uemg.br (G.M.); milena.1665493@discente.uemg.br (M.F.d.O.); leonardo.1665487@discente.uemg.br (L.M.G.R.); carlucio.1656958@discente.uemg.br (C.G.R.F.); 2 Laboratory for Research in Metabolism, Physiology, Physical Exercise, State University of Minas Gerais, Divinópolis 35501-170, Brazil; camila.brandao@uemg.br; 3 Research Group on Exercise, Sports Physiology, State University of Minas Gerais, Divinópolis 35501-170, Brazil; lucas.drummond@uemg.br; 4 Study and Research Group on Strength Training in Health, Physical Conditioning, State University of Minas Gerais, Divinópolis 35501-170, Brazil; lucas.lacerda@uemg.br; 5 Research Center for Psychological Assessment and Health, State University of Minas Gerais, Divinópolis 35501-170, Brazil; michelle.pereira@uemg.br

**Keywords:** visual attention, eye tracking, social cognition, child development, facial emotions

## Abstract

**Highlights:**

**What are the main findings?**

- Eye-tracking measures revealed distinct visual attention patterns toward emotional facial expressions in children, particularly for socially relevant facial regions such as the eyes and mouth.
- Gaze-based indicators demonstrated sensitivity to emotional intensity and social-emotional processing.

**What are the implications of the main findings?**

- Eye-tracking paradigms may contribute to the early identification of atypical socio-emotional processing associated with neurodevelopmental and psychiatric conditions in childhood.
- The integration of visual attention metrics with emotional recognition tasks provides a promising framework for advancing translational research in developmental neuroscience and pediatric mental health.

**Abstract:**

Objectives: Visual attention to emotional faces provides a useful framework for investigating orienting, visual exploration, and attentional engagement across development. The present study aimed to characterize the visuospatial organization of attention in neurotypical children and to examine how this pattern is modulated by social and emotional factors. Twenty children (aged 8–12 years) participated in a passive viewing paradigm of facial expressions while their eye movements were recorded using eye tracking (120 Hz). Methods: Oculomotor metrics based on areas of interest (eyes, mouth, nose, face, and non-social regions) were analyzed, including time to first fixation (TTFF), number of fixations (NF), and total fixation duration (TFD), as well as total saccade count as a global index of visual scanning. Results: Results indicated statistically significant AOI-dependent interactions involving emotional expression, observer sex, stimulus sex, and stimulus race/ethnicity, revealing region-specific modulation of visual attention. Consistently, prioritization of the eye region was observed, particularly for angry expressions, and was associated with greater fixation recurrence and duration, whereas happy and surprised expressions were associated with increased attentional allocation to the mouth. Differences related to observer sex and stimulus characteristics reflected region-specific modulations. In contrast, global saccadic dynamics remained relatively stable across experimental conditions and showed no significant effects of observer sex, stimulus sex, race/ethnicity, or emotional expression. Conclusions: Taken together, these findings suggest that visual attention to emotional faces in childhood follows a relatively stable spatial organization characterized by preferential processing of the eye region and region-specific modulation associated with emotional expression and stimulus characteristics.

## 1. Introduction

Social attention to emotional faces constitutes a central component of human social cognition, supporting processes such as recognition of affective states, inference of intentions, and regulation of interpersonal behavior. Despite advances in the field, the way visual attention is organized during face processing in childhood, particularly with respect to its modulation by sex and ethnicity, remains insufficiently characterized. This gap is relevant, as gaze allocation patterns reflect perceptual and social mechanisms that are fundamental for the interpretation of human stimuli.

During typical development, visual social cognition emerges from the interaction between neurobiological maturation and social experience. From the first months of life, children show preferential orientation to faces and sensitivity to emotional cues, with these processes progressively refined across childhood [1,2]. This refinement has been described by the broad-to-differentiated hypothesis, according to which initially broad emotional categories become more specific and culturally modulated over development [3,4]. At the neural level, circuits involving the amygdala and cortical regions associated with social perception contribute to the prioritization of emotionally relevant stimuli, especially human faces, facilitating gaze orientation toward highly informative facial regions such as the eyes [5,6,7,8].

Visual attention to faces can be described in terms of the spatial organization of gaze behavior, operationalized through the allocation of fixations to Areas of Interest (AOIs), such as the eyes, mouth, nose, and the face as a whole. This approach allows for the identification of which facial regions are prioritized during emotional processing, providing direct indices of visual exploration and attentional engagement. Although the temporal dimension of visual attention includes more complex dynamic aspects—such as scan paths and saccadic or antisaccadic transitions between regions—eye-tracking studies enable the capture of temporal components of attention and working visual memory through measures such as number of fixations, latency to first fixation, and total fixation duration. These indices reflect mechanisms of initial orienting, sustained attention, and working memory throughout stimulus exposure [1,9,10].

Beyond neurobiological mechanisms, the organization of social attention is modulated by social and cultural factors [11]. Variables such as observer sex and social characteristics of facial stimuli, including sex and ethnicity, influence patterns of visual attention and emotion recognition. Evidence suggests that these factors may shape perceptual biases and the way social information is extracted from faces, reflecting both biological predispositions and experience-dependent learning [12]. Phenomena such as the ingroup advantage and cultural differences in the prioritization of facial regions illustrate how visual social cognition emerges from an integrated system combining attentional neural circuits with cultural norms and communicative practices [11]. Nevertheless, systematic characterization of these modulations in typically developing children remains limited, especially in populations with high ethnic diversity.

In this context, eye tracking emerges as a robust methodological tool for investigating social visual attention in children, allowing for continuous and noninvasive measurement of visual exploration patterns during face processing. This approach provides direct access to implicit processes of attentional orienting and engagement, independent of verbal responses or explicit cognitive demands [9,13]. Measures such as orienting latency, fixation duration, and fixation count provide sensitive indicators of the spatial distribution of gaze and discrete temporal components associated with visual attention. These measures are particularly useful in socioculturally diverse contexts, such as Brazil, where linguistic and educational differences may introduce biases in traditional paradigms of culture and ethnicity.

Given this framework, the primary objective of the present study is to characterize the visuospatial behavior of visual attention across AOIs of emotional facial expressions in children aged 8 to 12 years. Specifically, the study aims to investigate how attention is distributed between the global face and informative facial regions during emotional categorization. As a secondary objective, the study examines the modulatory effects of social characteristics of facial stimuli (sex and ethnicity), as well as their interactions with different emotional valences, contributing to a more integrated understanding of the neurocognitive and sociocultural determinants of visual social cognition in childhood.

## 2. Materials and Methods

### 2.1. Participants

A total of 20 children (12 boys and 8 girls), aged 8 to 12 years (M = 9.8, SD = 1.12), participated in this study. Participants were recruited through convenience sampling via advertisements on local social media platforms. The children were enrolled between the 3rd and 8th grades of elementary school and had no history of grade retention. None of the participants had previously taken part in clinical or research studies involving facial expression stimuli or eye-tracking procedures. Participation was voluntary and conducted in accordance with the principles of the Declaration of Helsinki. The study was approved by the Research Ethics Committee of the Universidade do Estado de Minas Gerais (Approval No. 52539221.4.0000.5115). Neurotypical development was confirmed through standardized behavioral screening using the Social Responsiveness Scale–Second Edition (SRS-2).

### 2.2. Measures

Sociodemographic Questionnaire. A questionnaire developed for the present study was used to characterize the sample in terms of sociodemographic variables (age, sex, years of formal education) and general clinical information related to child development.

NEPSY–II. The NEPSY–II is a comprehensive neuropsychological assessment battery for children and adolescents aged 3 to 16 years. It comprises 32 subtests organized into six domains. In the present study, three specific subcomponents were used: Memory for Faces (MF), which assesses the ability to encode and recognize previously presented faces, serving as a measure of visual working memory; Affect Recognition (AR), which evaluates the ability to identify and discriminate basic emotions from facial expressions, reflecting perceptual and sociocognitive processes; and Theory of Mind (ToM), which includes verbal and contextual tasks that assess the understanding of one’s own mental states and the attribution of mental states to others.

Social Responsiveness Scale–Second Edition (SRS-2). This instrument was used as a screening tool to assess sociocommunicative traits associated with autism spectrum disorder (ASD). The scale consists of 65 items and can be completed by parents, teachers, or the participant (when applicable), covering domains such as social awareness, social cognition, social communication, social motivation, restricted/repetitive behaviors, and social communication and interaction. The Brazilian version presents evidence of validity and reliability and is appropriate for different age groups and administration formats (paper or digital [14]. Interventionary studies involving animals or humans, and other studies that require ethical approval, must list the authority that provided approval and the corresponding ethical approval code.

### 2.3. Apparatus

A Tobii eye-tracking system (Tobii Technology, Danderyd, Sweden) was used to record participants’ oculomotor activity during the viewing of emotional facial expression stimuli. The system operated at a sampling rate of 120 Hz and was integrated with a 23-inch monitor (1920 × 1080 pixels), on which the stimuli were presented. Calibration was performed using an eight-point procedure distributed across the visual field of the screen. Calibration was considered acceptable when it achieved an angular error ≤ 1.0° and precision ≤ 0.5°, in accordance with the manufacturer’s technical recommendations. This procedure was conducted prior to the experimental task. Stimulus presentation, as well as data acquisition and analysis, were carried out using Tobii Studio™ software (version 3.4.0, Tobii Technology, Sweden).

### 2.4. Procedure and Experimental Paradigm

A mixed-design study with a factorial structure was employed, integrating neuropsychological assessment and eye-tracking measures. Initially, 37 participants were enrolled in the study, of whom 29 began the data collection phase. During the process, nine participants withdrew for personal reasons, resulting in the final analyzed sample. All procedures were conducted at the Laboratory of Neuroscience, Chronobiology, and Sleep Psychology (LNCPs) in two experimental sessions. In the initial phase, sociodemographic information was collected, and clinical and neuropsychological assessments were conducted to determine eligibility. Only participants who met the inclusion criteria proceeded to the experimental phase.

The experimental task consisted of a passive viewing paradigm of emotional facial expressions, in which participants freely observed the stimuli without the requirement for explicit behavioral responses, while eye movements were continuously recorded.

**Figure 1:**
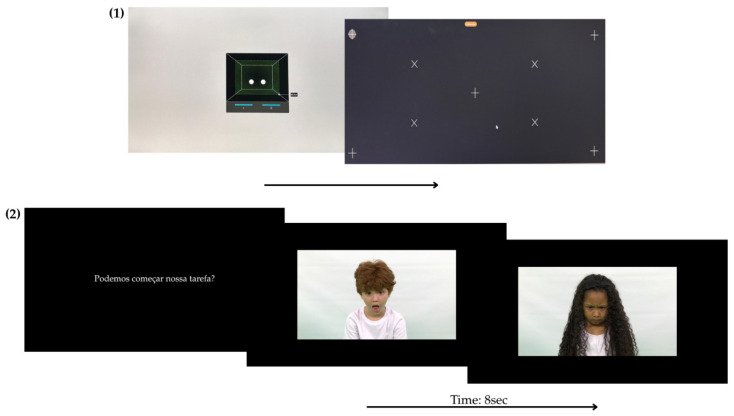
Schematic representation of the experimental sequence used in the eye-tracking task. After distance adjustment and calibration (**1**), participants viewed emotional facial expressions presented individually in a passive viewing paradigm (**2**). Stimuli included different emotional categories and facial characteristics, while binocular eye movements were continuously recorded to evaluate patterns of visual attention during face processing.

Following system calibration, an instructional screen was presented to the participant with the prompt: “*Can we start our task*?” This instruction aimed at orienting the child to the beginning of the task. A total of 56 facial stimuli were used, selected from the database [15], developed specifically for research on emotional recognition in children. The stimuli were presented individually and centrally on the screen for 8 s each, following a pseudo-randomized sequence balanced across eight emotional categories (neutral, happiness, surprise, anger, sadness, disgust, contempt, and fear) and across the ethnicity of the facial models, in order to minimize order effects, perceptual adaptation, and frequency biases (Figure 1).

The total presentation time was 7.47 min, during which children were instructed to freely observe the faces without providing additional responses. Eye movements were recorded binocularly throughout the entire task. Assessments were conducted in a quiet environment with controlled lighting and minimal external interference. Participants were seated comfortably in a fixed chair at a distance of 60 cm from the monitor, with the screen positioned at eye level and without the use of head stabilization devices. Oculometric recording was performed binocularly.

### 2.5. Study Variables

**Figure 2:**
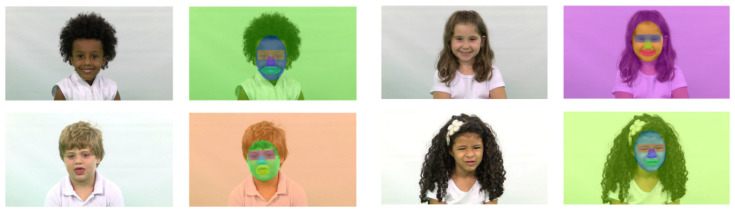
Examples of facial stimuli obtained from the ChildEFES database and schematic representation of the predefined Areas of Interest (AOIs) used for eye-movement analyses. AOIs included the eyes, mouth, nose, whole face, and non-social regions outside the facial area. Oculometric measures extracted from these regions comprised time to first fixation (TTFF), number of fixations (NF), and total fixation duration (TFD), allowing for the characterization of attentional orienting, visual exploration, and sustained attention during emotional face processing.

The adopted design allowed for the examination of both normative patterns of social visual attention and the modulation of these patterns as a function of the social characteristics of the stimuli. The independent variables explicitly included observer sex, as well as facial stimulus characteristics, specifically stimulus sex, race/ethnicity, and emotional expression. The dependent variables consisted of predefined oculometric metrics based on the delineation of Areas of Interest (AOIs). The following AOIs were defined: eyes (AOI–Eyes), mouth (AOI–Mouth), nose (AOI–Nose), whole face (AOI–Face), and non-social regions of the image (AOI–Non-social), corresponding to areas outside the facial region (Figure 2).

### 2.6. Data Analysis

Data were organized in spreadsheets and analyzed using IBM SPSS Statistics (Version 24). Normality of distributions was assessed using the Shapiro–Wilk test, and homogeneity of variances was evaluated with Levene’s test. To account for the hierarchical structure of the eye-tracking data, linear mixed-effects models fitted by restricted maximum likelihood (REML) were employed. Participant was included as a random intercept, whereas observer sex, stimulus sex, stimulus race/ethnicity, emotional expression, and area of interest (AOI), as well as their interactions, were specified as fixed effects. Type III tests of fixed effects were used to evaluate statistical significance. The significance level was set at *p* < 0.05, and Bonferroni corrections were applied for multiple comparisons. Effect sizes were estimated using partial eta squared (η^2^p).

Because multiple observations were obtained from each participant across different facial stimuli and AOIs, the use of mixed-effects models allowed the hierarchical structure of the data to be appropriately modeled while accounting for within-subject dependencies. Estimated marginal means (EMMs) and Bonferroni-adjusted pairwise comparisons were used to further characterize significant interactions.

### 2.7. Extraction and Processing of Oculomotor Measures

Eye movement metrics were extracted using the I-VT (Velocity-Threshold Identification) algorithm, adopting a velocity threshold of 30°/s and a minimum fixation duration of 60 ms. Trials with less than 90% valid samples were excluded to ensure data quality. Oculometric measures were organized into functional classes: indices of initial attentional orienting, operationalized as time to first fixation on the eye region, considered indicators of early attentional prioritization of socially relevant cues; indices of sustained visual attention, represented by fixation count and total fixation duration within each AOI, used to characterize the allocation and maintenance of attention throughout stimulus exploration; and indices of dynamic visual exploration, estimated by the number of saccades between social AOIs, reflecting patterns of visual scanning and integration of facial information.

## 3. Results

### 3.1. Sociodemographic and Neuropsychological Data

In the neuropsychological assessment, groups showed similar performance across most investigated domains. In the MF subtest, a significant sex difference was observed (U = 22; Z = −2.05; *p* = 0.047), with higher performance in the female group, M = 25.25 (SD = 1.16; Md = 25.0; 95% CI [24.28–26.22]), compared to boys, M = 23.27 (SD = 2.52; Md = 23.0; 95% CI [21.67–24.87]). Although girls obtained significantly higher scores on the Memory for Faces subtest (U = 22; Z = −2.05; *p* = 0.047), ROC curve analysis indicated poor discriminative performance at the individual level (AUC = 0.229; *p* = 0.045), suggesting substantial overlap between the score distributions of boys and girls. Therefore, while a group-level difference was detected, the measure showed limited ability to accurately classify participants according to sex.

For the remaining domains, no statistically significant differences were observed. In the AR subtest (*p* = 0.129), boys showed M = 43.73 (SD = 27.28; Md = 37.0; 95% CI [26.41–61.05]), whereas girls showed M = 64.38 (SD = 26.37; Md = 63.0; 95% CI [42.33–86.43]). For ToM, mean scores were M = 65.67 (SD = 20.83; Md = 63.0; 95% CI [52.43–78.91]) for boys and M = 81.50 (SD = 15.28; Md = 84.0; 95% CI [68.72–94.28]) for girls (*p* = 0.098). Similarly, SRS-2 scores did not differ between groups (Boys: M = 49.00; SD = 10.50; Md = 53.0; 95% CI [42.33–55.67]; Girls: M = 46.25; SD = 4.77; Md = 46.0; 95% CI [42.26–50.24]; *p* = 0.098).

### 3.2. Eye Movements

#### 3.2.1. Orienting Visual Attention

Linear mixed-effects analyses revealed significant two-way interactions between observer sex and stimulus race/ethnicity, F(1, 4274) = 3.95, *p* = 0.047, η^2^p = 0.001, stimulus sex and area of interest (AOI), F(4, 4274) = 6.70, *p* < 0.001, η^2^p = 0.006, stimulus race/ethnicity and AOI, F(4, 4274) = 5.17, *p* < 0.001, η^2^p = 0.005, and emotion and AOI, F(28, 4274) = 4.98, *p* < 0.001, η^2^p = 0.032 (Table 1). No significant interactions were observed for observer sex × stimulus sex, observer sex × emotion, observer sex × AOI, stimulus sex × race/ethnicity, stimulus sex × emotion, or race/ethnicity × emotion (all ps > 0.05) (Table 1).

At the three-way level, the observer sex × emotion × AOI interaction was not significant, F(28, 4274) = 0.75, *p* = 0.824, η^2^p = 0.005. In contrast, significant interactions emerged for stimulus sex × emotion × AOI, F(28, 4274) = 2.34, *p* < 0.001, η^2^p = 0.015, and race/ethnicity × emotion × AOI, F(28, 4274) = 2.39, *p* < 0.001, η^2^p = 0.015. Furthermore, a significant four-way interaction involving stimulus sex × race/ethnicity × emotion × AOI was observed, F(20, 4274) = 2.76, *p* < 0.001, η^2^p = 0.013. Neither the observer sex × race/ethnicity × emotion × AOI interaction, F(32, 4274) = 1.24, *p* = 0.165, η^2^p = 0.009, nor the observer sex × stimulus sex × race/ethnicity × emotion interaction, F(12, 4274) = 0.62, *p* = 0.824, η^2^p = 0.002, reached statistical significance. Likewise, the five-way interaction among observer sex, stimulus sex, race/ethnicity, emotion, and AOI was not significant, F(52, 4274) = 1.15, *p* = 0.212, η^2^p = 0.014.

Bonferroni-adjusted pairwise comparisons based on estimated marginal means (EMMs) indicated that early attentional orienting was primarily modulated by the interaction between emotional expression and facial regions. Estimated marginal means for the significant Emotion × AOI interaction are presented in Supplementary Table S1A. Across emotions, the whole-face AOI consistently exhibited the shortest TTFF values, whereas non-social regions showed the longest fixation latencies. Emotional effects were particularly pronounced for socially informative regions. Specifically, angry faces elicited the shortest orienting latencies toward the eye region (M = 414.52 ms, SE = 152.86), whereas surprise and contempt expressions were associated with comparatively longer latencies (Supplementary Table S1A; Figure 1). In addition, anger and fear were characterized by prolonged orienting toward the mouth and nose regions relative to other emotional expressions.

The significant stimulus sex × emotion × AOI interaction further indicated that the spatial allocation of early attention varied between female and male faces depending on the emotional expression displayed (Figure 2). Estimated marginal means for this interaction are reported in Supplementary Table S1B. Overall, female faces expressing anger were associated with particularly rapid orienting toward the eyes and longer latencies toward the mouth and nose regions. In contrast, male faces exhibited distinct emotion-dependent attentional profiles, especially for the nose and non-social regions.

Likewise, the significant race/ethnicity × emotion × AOI interaction demonstrated that emotional information was differentially extracted from facial regions as a function of stimulus race/ethnicity. Estimated marginal means are presented in Supplementary Table S1C. Although both White and Black faces showed emotion-specific attentional patterns, differences were especially evident for the eye, mouth, and non-social regions, suggesting that race/ethnicity modulated the spatial distribution of visual attention during the earliest stages of face processing.

Taken together, these findings indicate that early attentional orienting was primarily determined by the interaction between emotional expression and specific facial regions, with additional modulation by stimulus sex and race/ethnicity. In contrast, observer sex exerted only a limited influence and did not contribute significantly to higher-order interactions. Thus, the mechanisms underlying initial visual attention appear to be driven predominantly by characteristics of the facial stimulus rather than by characteristics of the observer.

#### 3.2.2. Visual Exploration and Oculomotor Dynamics

##### Number of Fixations (NF)

As shown in Table 2, mixed-effects analyses revealed significant two-way interactions involving observer sex × AOI, stimulus sex × emotion, stimulus sex × AOI, race/ethnicity × AOI, and emotion × AOI. No higher-order interactions reached significance (Supplementary Table S1).

A significant observer sex × AOI interaction indicated that fixation allocation differed across facial regions as a function of participant sex, F(4, 5190) = 22.69, *p* < 0.001, η^2^p = 0.017. Across both sexes, the highest fixation counts were observed for the whole-face AOI and the eye region, whereas the mouth and nose received substantially fewer fixations. This pattern suggests that visual exploration was preferentially directed toward socially informative facial features.

Stimulus sex also modulated visual exploration. A significant stimulus sex × AOI interaction, F(4, 5190) = 5.82, *p* < 0.001, η^2^p = 0.004, indicated that the distribution of fixation counts varied according to whether the presented face was male or female. Moreover, a significant stimulus sex × emotion interaction, F(7, 5190) = 2.32, *p* = 0.023, η^2^p = 0.003, revealed that emotional effects differed as a function of stimulus sex, suggesting that the processing of facial expressions was influenced by whether the face belonged to a male or female individual.

A significant race/ethnicity × AOI interaction was also observed, F(4, 5190) = 4.29, *p* = 0.002, η^2^p = 0.003, indicating that the distribution of gaze across facial regions differed according to stimulus race/ethnicity. However, no significant race/ethnicity × emotion interaction was found, F(7, 5190) = 0.63, *p* = 0.728, suggesting that emotional expressions elicited comparable fixation patterns across White and Black faces. Estimated marginal means showed only minor numerical differences between racial groups. For both White and Black faces, surprise expressions were associated with the highest overall fixation counts (White: M = 6.08, 95% CI [5.72, 6.44]; Black: M = 5.85, 95% CI [5.37, 6.34]), whereas sadness produced the lowest values (White: M = 5.35, 95% CI [5.00, 5.69]; Black: M = 5.31, 95% CI [4.89, 5.73]).

Emotional expression significantly influenced fixation distribution across AOIs, as evidenced by a significant emotion × AOI interaction, F(28, 5190) = 3.32, *p* < 0.001, η^2^p = 0.018. Across all emotional expressions, the whole-face AOI consistently received the highest number of fixations, followed by the eye region. Angry faces elicited the greatest number of fixations to the eyes (M = 8.14, 95% CI [7.49, 8.78]), followed by neutral (M = 7.79, 95% CI [7.19, 8.38]) and fearful expressions (M = 7.63, 95% CI [6.98, 8.27]). In contrast, happy (M = 6.21, 95% CI [5.56, 6.85]) and sad faces (M = 6.64, 95% CI [6.04, 7.25]) elicited comparatively fewer fixations to the eye region. In the mouth AOI, surprise (M = 2.83, 95% CI [2.20, 3.46]), happiness (M = 2.67, 95% CI [2.02, 3.32]), and disgust (M = 2.40, 95% CI [1.75, 3.04]) produced relatively higher fixation counts, whereas anger elicited the lowest values (M = 0.91, 95% CI [0.26, 1.56]). The non-social AOI showed increased exploration for angry (M = 3.87, 95% CI [3.22, 4.52]) and contemptuous faces (M = 3.81, 95% CI [3.22, 4.41]), whereas disgust was associated with the lowest non-social fixation counts (M = 2.42, 95% CI [1.77, 3.07]). Finally, surprised faces elicited the highest number of fixations to the whole-face AOI (M = 14.33, 95% CI [13.60, 15.05]), whereas contempt (M = 12.79, 95% CI [12.19, 13.38]) and sadness (M = 12.76, 95% CI [12.15, 13.36]) produced comparatively lower values.

No significant three-, four-, or five-way interactions were detected (all ps > 0.05), indicating that the effects of emotion and facial region on visual attention were relatively stable across observer sex, stimulus sex, and race/ethnicity.

##### Total Fixation Duration (TFD)

As shown in Table 2, mixed-effects analyses revealed that total fixation duration (TFD) varied as a function of area of interest (AOI)-dependent effects. Significant interactions were observed for observer sex × AOI, F(4, 5190) = 44.28, *p* < 0.001, η^2^p = 0.033, stimulus sex × AOI, F(4, 5190) = 5.45, *p* < 0.001, η^2^p = 0.004, race/ethnicity × AOI, F(4, 5190) = 11.44, *p* < 0.001, η^2^p = 0.009, emotion × AOI, F(28, 5190) = 4.92, *p* < 0.001, η^2^p = 0.026, and stimulus sex × emotion × AOI, F(28, 5190) = 1.66, *p* = 0.016, η^2^p = 0.009.

Bonferroni-adjusted pairwise comparisons revealed robust AOI-dependent effects. Female participants exhibited longer fixation durations in socially informative facial regions, including the eyes, mouth, nose, and the face as a whole, whereas male participants devoted more viewing time to non-social regions. Across both sexes, the eye and whole-face AOIs consistently attracted the greatest visual engagement, indicating preferential maintenance of attention toward socially relevant facial information.

Stimulus sex also modulated sustained visual engagement, as evidenced by the significant stimulus sex × AOI interaction. Male faces elicited longer fixation durations in the eye and whole-face regions, whereas female faces were associated with relatively greater exploration of non-social regions. Likewise, the significant race/ethnicity × AOI interaction indicated that the effects of race/ethnicity depended on facial region. White faces elicited longer fixation durations in the eye and whole-face AOIs, whereas Black faces were associated with greater engagement with the mouth and non-social regions.

Emotional expression was a major determinant of fixation duration across facial regions, as indicated by the significant emotion × AOI interaction. In the eye region, angry expressions elicited the longest fixation durations, reflecting enhanced attentional maintenance toward ocular cues associated with threat-related information. In contrast, emotional modulation in the mouth region followed an opposite pattern, with happy, disgusted, neutral, and contempt expressions eliciting greater fixation durations than angry expressions. Effects in the nose region were comparatively modest. In the non-social AOI, disgust was associated with the lowest fixation durations, whereas anger and contempt were linked to increased visual exploration outside facial regions. Emotional differences in the whole-face AOI were comparatively smaller, suggesting that emotional expression primarily influenced the distribution of attention across facial subregions rather than overall visual engagement.

Importantly, the significant stimulus sex × emotion × AOI interaction indicated that the effects of emotional expression on fixation duration differed according to the sex of the face being viewed. Thus, emotional modulation of sustained visual engagement was not uniform across male and female faces, suggesting a complex interplay between emotional content and stimulus sex in directing visual attention.

##### Attentional Engagement and Visual Exploration

NF and TFD revealed largely convergent patterns of visual attention allocation, suggesting that these metrics capture complementary aspects of attentional engagement during face processing (Table 2). Across all conditions, the whole-face and eye AOIs consistently received the highest fixation counts and longest fixation durations, indicating that regions that were revisited most frequently were also those in which gaze was maintained for longer periods. In contrast, the mouth, nose, and non-social regions received comparatively less visual attention.

For both metrics, visual attention was primarily modulated by AOI-dependent effects. Significant interactions involving AOI were observed for observer sex, stimulus sex, race/ethnicity, and emotional expression, indicating that the influence of these factors varied according to facial region. Female participants exhibited greater visual engagement with socially informative regions, whereas male participants devoted relatively more attention to non-social areas. Likewise, male faces elicited greater engagement with the eyes and whole-face regions, whereas female faces were associated with increased attention to non-social regions. White faces attracted relatively greater visual engagement with the eyes and whole face, whereas Black faces elicited increased exploration of the mouth and non-social regions.

Emotional expression emerged as a major determinant of gaze distribution across facial regions. For both NF and TFD, angry expressions elicited the greatest fixation counts and longest fixation durations in the eye region, followed by neutral and fearful expressions, suggesting enhanced attentional prioritization of ocular cues associated with threat-related information. By contrast, the mouth region exhibited the opposite pattern, with happy, disgusted, and surprised expressions producing greater visual engagement than angry expressions. Exploration of non-social regions was greatest for angry and contemptuous faces and lowest for disgusted faces, whereas emotional differences in the whole-face AOI were comparatively smaller. Together, these findings indicate that emotional information primarily influenced the allocation of attention across facial subregions rather than overall visual engagement.

Although NF and TFD showed highly similar spatial patterns, TFD revealed a more complex organization of emotional effects. Specifically, a significant stimulus sex × emotion × AOI interaction, F(28, 5190) = 1.66, *p* = 0.016, η^2^p = 0.009, indicated that the influence of emotional expressions on sustained attentional engagement differed according to the sex of the face being viewed. In contrast, no higher-order interactions were detected for fixation counts, suggesting that the spatial organization of visual exploration was relatively stable across observer sex, stimulus sex, and race/ethnicity.

Taken together, these findings suggest that visual attention to emotional faces is characterized by a common spatial organization across metrics, with preferential processing of the eyes and whole-face regions and emotion-specific modulation of gaze allocation across facial subregions. Whereas fixation counts primarily reflected the spatial distribution and recurrence of gaze, fixation duration additionally captured sustained attentional engagement, revealing a more nuanced interaction between emotional expression and stimulus sex.

#### 3.2.3. Dynamics of Visual Processing

Dynamic visual processing was indexed by the total number of saccades generated during the presentation of each facial stimulus (Table 3). Mixed-effects analyses revealed no significant interactions involving observer sex, stimulus sex, race/ethnicity, or emotional expression, indicating that global visual scanning behavior remained stable across experimental conditions.

## 4. Discussion

The findings of the present study suggest that visual attention to emotional faces in neurotypical children aged 8 to 12 years follows a relatively stable spatial pattern characterized by preferential allocation of attention to socially informative facial regions, particularly the eyes. This pattern converges with previous eye-tracking evidence identifying the eye region as the primary source of social and emotional information [16].

The integrated analysis of oculometric metrics—time to first fixation (TTFF), number of fixations (NF), and total fixation duration (TFD)—indicates that these indices reflect complementary components of processes underlying visual social attention. Taken together, the findings are compatible with regional redistribution of attention across facial areas rather than generalized increases in visual exploration [17]. Moreover, the significant Emotion × AOI interactions observed for both fixation counts and total fixation duration indicate that emotional modulation was primarily expressed through changes in the spatial allocation of attention across facial subregions. This interpretation is also consistent with evidence linking attentional orienting and oculomotor control. Studies have shown that shifts in visual attention are closely associated with the neural and behavioral mechanisms involved in saccade preparation and execution, including covert attentional orienting that may occur even in the absence of overt eye movements. Therefore, fixation patterns and saccadic behavior can be interpreted as behavioral indicators of attentional allocation during visual exploration of socially relevant stimuli [18].

This profile is consistent with developmental studies reporting early and relatively stable prioritization of the eye region throughout childhood [1,15,19]. However, the present data do not allow for inferences regarding the underlying developmental mechanisms [18,19].

Emotional expression was one of the variables most consistently associated with variations in visual attention distribution. Angry expressions were associated with greater recurrence and longer fixation duration in the eye region, whereas emotions such as happiness and surprise showed increased attentional allocation to the mouth region. This pattern is consistent with models proposing adaptive prioritization of ocular cues in contexts of higher social relevance or threat [20,21]. Behavioral evidence indicates that negative emotions, particularly anger, are recognized more accurately even at subtle levels [22], whereas different emotions recruit distinct perceptual strategies [23]. Taken together, the findings are compatible with regional redistribution of attention across facial areas rather than generalized increases in visual exploration [17]. Furthermore, the significant Emotion × AOI interactions observed for both FC and TFD indicate that emotional modulation was primarily expressed through changes in the spatial allocation of attention across facial subregions rather than through global increases in visual engagement.

Differences related to observer sex were observed in patterns of visual exploration, although no differences were detected in explicit neuropsychological measures. Girls showed greater engagement in socially relevant facial regions, whereas boys demonstrated greater exploration of non-social regions. This pattern is consistent with evidence that eye tracking captures implicit aspects of social attention and visual exploration [24] and with studies suggesting that sex-related differences reflect distinct exploratory styles rather than differences in socioemotional competence [25]. Importantly, these differences emerged mainly through interactions with AOIs, suggesting that sex-related effects reflect differences in gaze allocation strategies rather than generalized differences in visual attention.

Stimulus race/ethnicity was associated with small variations in visual attention allocation across facial and non-facial regions. These effects were limited in magnitude and may reflect characteristics of the presented stimuli. Because participant race/ethnicity was not examined as an analytical factor, the present findings do not allow for conclusions regarding cross-ethnic perception, in-group bias, or other observer-related sociocultural processes [26]. Therefore, interpretations concerning sociocultural influences should be considered preliminary [10,27]. Moreover, the small effect sizes associated with race/ethnicity-related interactions suggest that these influences were subtle and largely restricted to region-specific patterns of attention.

Although fixation counts and total fixation duration exhibited highly convergent patterns, TFD revealed a more complex organization of emotional effects. Specifically, the significant Stimulus Sex × Emotion × AOI interaction observed only for TFD suggests that sustained attentional engagement may be more sensitive than fixation frequency to subtle interactions between emotional content and stimulus characteristics. This finding highlights the importance of combining multiple oculometric indices to characterize social attention and suggests that fixation duration captures additional aspects of emotional processing beyond those reflected in fixation frequency alone.

In contrast to fixation-based measures, total saccade count remained remarkably stable across experimental conditions. No significant interactions involving observer sex, stimulus sex, race/ethnicity, or emotional expression were observed, suggesting that global visual scanning behavior was less sensitive to social and affective manipulations than fixation-based indices. This finding supports the notion that fixation measures provide a more sensitive characterization of social attention than overall oculomotor activity [17,24].

From a theoretical perspective, previous neuroimaging studies have associated emotional face processing with interactions between attentional and affective neural systems [28]. However, the present study did not include neural or physiological measures. Therefore, neurobiological interpretations remain speculative and should be considered only as a conceptual framework for understanding the observed behavioral patterns [16].

The relevance of these findings becomes particularly evident when contrasted with clinical populations. Studies involving autism spectrum disorder (ASD) and attention-deficit/hyperactivity disorder (ADHD) report reduced allocation of attention to the eye region, accompanied by alterations in initial orienting and sustained engagement [28,29,30,31]. In contrast, the present results indicate prioritization of the eye region, a pattern commonly reported in studies of typical development. Additionally, evidence indicates that relational factors, such as attachment security, are associated with modulation of emotional processing at basic perceptual levels [23]. Although not directly assessed, the present study provides a useful normative framework for future investigations integrating contextual and relational variables.

Some limitations should be considered. The small sample size and cross-sectional design limit generalizability and causal inference. Furthermore, the unequal distribution of participants across certain categories, particularly with respect to race/ethnicity, requires caution in interpreting the observed effects. Although linear mixed-effects models were employed to account for the hierarchical structure of the data, the relatively small sample size and the imbalance across some experimental categories may have limited statistical power to detect subtle interaction effects. Future studies using larger and more balanced samples may provide greater sensitivity for identifying individual differences in visual attention and emotional face processing.

## 5. Conclusions

Taken together, the findings suggest that visual attention to emotional faces in childhood follows a relatively stable spatial organization characterized by preferential processing of socially informative facial regions, particularly the eyes, and by region-specific modulation associated with emotional expression and stimulus characteristics. The integration of multiple oculometric metrics, including time to first fixation, fixation counts, and total fixation duration, provided a comprehensive characterization of complementary components of social attention, revealing that emotional effects were primarily expressed through changes in the spatial allocation of attention across facial subregions rather than through generalized increases in visual engagement. Moreover, the greater sensitivity of total fixation duration to the interaction between emotional expression and stimulus sex highlights the importance of combining multiple oculometric indices when investigating social attention. Overall, these findings provide normative evidence regarding patterns of visual attention to emotional faces in Brazilian children and may contribute to future investigations of social attention in both typical and atypical development.

## Figures and Tables

**Table 1:** Fixed effects of factors and interactions on time to first fixation (TTFF) derived from linear mixed-effects models.

Factor/Interaction	F	Num df	Den df	*p*	η^2^p
Two-way interactions					
Observer sex × Stimulus sex	1.735	1	4274	0.188	0.000
Observer sex × Race/ethnicity	3.945	1	4274	0.047 *	0.001
Observer sex × Emotion	1.297	7	4274	0.247	0.002
Observer sex × AOI	2.110	4	4274	0.077	0.002
Stimulus sex × Race/ethnicity	2.141	1	4274	0.144	0.001
Stimulus sex × Emotion	1.321	7	4274	0.236	0.002
Stimulus sex × AOI	6.701	4	4274	<0.001 *	0.006
Race/ethnicity × Emotion	0.568	7	4274	0.782	0.001
Race/ethnicity × AOI	5.165	4	4274	<0.001 *	0.005
Emotion × AOI	4.984	28	4274	<0.001 *	0.032
Three-way interactions					
Observer sex × Emotion × AOI	0.751	28	4274	0.824	0.005
Stimulus sex × Emotion × AOI	2.338	28	4274	<0.001 *	0.015
Race/ethnicity × Emotion × AOI	2.391	28	4274	<0.001 *	0.015
Four-way interactions					
Observer sex × Race/ethnicity × Emotion × AOI	1.242	32	4274	0.165	0.009
Stimulus sex × Race/ethnicity × Emotion × AOI	2.759	20	4274	<0.001 *	0.013
Observer sex × Stimulus sex × Race/ethnicity × Emotion	0.624	12	4274	0.824	0.002
Five-way interaction					
Observer sex × Stimulus sex × Race/ethnicity × Emotion × AOI	1.152	52	4274	0.212	0.014

Note. Fixed effects were evaluated using linear mixed-effects models fitted by restricted maximum likelihood (REML), including participant as a random intercept to account for repeated observations. F values correspond to Type III tests of fixed effects. * Statistical significance was defined as *p* < 0.05. Num df = numerator degrees of freedom; Den df = denominator degrees of freedom; η^2^p = partial eta squared.

**Table 2:** Significant interactions from linear mixed-effects models predicting fixation counts (FC) and total fixation duration (TFD).

Factor/Interaction	FC					TFD				
	F	Num df	Den df	*p*	η^2^p	F	Num df	Den df	*p*	η^2^p
Two-way interactions										
Observer sex × Stimulus sex	0.588	1	5190	0.443	0.000	0.024	1	5190	0.878	0.000
Observer sex × Race/ethnicity	0.050	1	5190	0.823	0.000	0.285	1	5190	0.594	0.000
Observer sex × Emotion	1.151	7	5190	0.328	0.002	0.839	7	5190	0.555	0.001
Observer sex × AOI	22.687	4	5190	<0.001	0.017	44.277	4	5190	<0.001	0.033
Stimulus sex × Race/ethnicity	0.196	1	5190	0.658	0.000	0.293	1	5190	0.588	0.000
Stimulus sex × Emotion	2.317	7	5190	0.023	0.003	0.670	7	5190	0.698	0.001
Stimulus sex × AOI	5.823	4	5190	<0.001	0.004	5.451	4	5190	<0.001	0.004
Race/ethnicity × Emotion	0.634	7	5190	0.728	0.001	0.575	7	5190	0.777	0.001
Race/ethnicity × AOI	4.286	4	5190	0.002	0.003	11.441	4	5190	<0.001	0.009
Emotion × AOI	3.324	28	5190	<0.001	0.018	4.920	28	5190	<0.001	0.026
Three-way interactions										
Observer sex × Emotion × AOI	0.582	28	5190	0.961	0.003	0.930	28	5190	0.570	0.005
Stimulus sex × Emotion × AOI	1.380	28	5190	0.088	0.007	1.656	28	5190	0.016	0.009
Race/ethnicity × Emotion × AOI	1.014	28	5190	0.444	0.005	1.222	28	5190	0.195	0.007
Four-way interactions										
Observer sex × Race/ethnicity × Emotion × AOI	0.774	32	5190	0.815	0.005	0.791	32	5190	0.793	0.005
Stimulus sex × Race/ethnicity × Emotion × AOI	0.837	20	5190	0.669	0.003	1.406	20	5190	0.107	0.005
Observer sex × Stimulus sex × Race/ethnicity × Emotion	1.000	12	5190	0.446	0.002	0.547	12	5190	0.885	0.001
Five-way interaction										
Observer sex × Stimulus sex × Race/ethnicity × Emotion × AOI	0.470	52	5190	1.000	0.005	0.574	52	5190	0.994	0.006

Note. FC = fixation counts; TFD = total fixation duration; AOI = area of interest. Results are based on Type III tests of fixed effects from linear mixed-effects models fitted using restricted maximum likelihood (REML). Num df = numerator degrees of freedom; Den df = denominator degrees of freedom; η^2^p = partial eta squared.

**Table 3:** Type III tests of fixed interactions for total saccade count obtained from linear mixed-effects models.

Factor/Interaction	F	Num df	Den df	*p*
Two-way interactions				
Observer sex × Stimulus sex	0.226	1	1062	0.634
Observer sex × Race/ethnicity	0.634	1	1062	0.426
Observer sex × Emotion	0.796	7	1062	0.590
Stimulus sex × Race/ethnicity	0.250	1	1062	0.617
Stimulus sex × Emotion	1.511	7	1062	0.159
Race/ethnicity × Emotion	0.407	7	1062	0.899
Three-way interactions				
Observer sex × Stimulus sex × Race/ethnicity	1.202	1	1062	0.273
Observer sex × Race/ethnicity × Emotion	0.225	7	1062	0.980
Stimulus sex × Race/ethnicity × Emotion	1.112	4	1062	0.349
Observer sex × Stimulus sex × Emotion	0.597	7	1062	0.759
Three-way interactions				
Observer sex × Stimulus sex × Race/ethnicity × Emotion	0.484	11	1062	0.914

Note. Results are based on Type III tests of fixed effects from linear mixed-effects models fitted using restricted maximum likelihood (REML). Num df = numerator degrees of freedom; Den df = denominator degrees of freedom.

## Data Availability

The original contributions presented in this study are included in the article. Further inquiries can be directed to the corresponding author.

## Supplementary Materials

The following supporting information can be downloaded at: https://www.mdpi.com/article/10.3390/brainsci16070683/s1, Table S1: Descriptive statistics for time to first fixation (TTFF) across the analyzed conditions; Table S2: Descriptive statistics for fixation counts (FC) and total fixation duration (TFD) across the analyzed conditions.
