# Supplementary material for: Visual Attention to Emotional Faces in Children: An Eye-Tracking Study of Social Visual Attention
Source: Brain Sci. 2026 Jun 29;16(7):683. doi: 10.3390/brainsci16070683 (PMC13406557; doi:10.3390/brainsci16070683)
Supplement: Supplementary file 1 [file brainsci-16-00683-s001.zip › brainsci-4356263-supplementary.pdf]

Supplementary Table S1

Supplementary Table S1A. Estimated marginal means (EMMs) for time to first fixation (TTFF, ms) according to emotional expression and area of interest (AOI).

Emotion	Eyes	Mouth	Nose	Whole Face	Non-social
**Contempt**	876.97 (139.33)	2277.82 (175.39)	1557.02 (149.29)	164.25 (137.32)	2509.59 (158.07)
**Happiness**	784.64 (158.63)	1263.89 (163.23)	1374.85 (157.53)	167.30 (150.13)	2992.15 (177.17)
**Fear**	583.62 (153.85)	1555.30 (181.78)	1987.16 (170.31)	129.22 (150.13)	2573.88 (183.71)
**Neutral**	712.78 (139.06)	2647.45 (179.51)	1336.53 (146.16)	145.54 (137.77)	2314.43 (157.21)
**Disgust**	806.53 (153.66)	1772.73 (167.11)	1541.97 (160.41)	118.79 (150.23)	3084.73 (183.51)
**Anger**	414.52 (152.86)	3502.79 (202.83)	2239.63 (178.16)	146.81 (150.36)	2509.40 (173.31)
**Surprise**	893.13 (148.77)	1461.22 (163.09)	1457.36 (155.68)	176.83 (168.18)	2526.54 (177.37)
**Sadness**	648.64 (144.52)	2377.99 (175.51)	1458.26 (154.14)	102.14 (141.02)	2643.37 (174.74)

Supplementary Table S1B. Estimated marginal means (EMMs) for time to first fixation (TTFF, ms) according to stimulus sex, emotional expression, and area of interest (AOI).

Female Faces

Emotion	Eyes	Mouth	Nose	Whole Face	Non-social
**Contempt**	893.6	2278.0	1592.4	158.5	2605.3
**Happiness**	970.9	753.8	1027.7	83.9	2775.9
**Fear**	699.6	1352.3	2205.7	171.9	2281.3
**Neutral**	754.5	2770.3	1119.5	157.0	1858.6
**Disgust**	859.5	2205.9	1410.1	101.5	2098.4
**Anger**	357.9	3766.1	2208.6	223.4	2218.2

**Surprise**	1045.5	1575.9	1260.7	232.3	2102.3
**Sadness**	807.1	2092.2	1407.4	139.1	2874.7

Male Faces

Emotion	Eyes	Mouth	Nose	Whole Face	Non-social
**Contempt**	765.1	2283.0	1633.3	175.8	3073.2
**Happiness**	598.4	1774.0	1722.0	250.7	3208.4
**Fear**	467.6	1766.3	1918.6	104.7	2860.2
**Neutral**	671.4	2401.7	1770.5	122.7	3226.2
**Disgust**	753.6	1339.6	1673.9	136.0	4071.1
**Anger**	471.1	3065.8	2270.6	70.2	2800.6
**Surprise**	588.3	1231.8	1850.7	65.9	3375.1
**Sadness**	490.1	2663.8	1509.1	65.2	2412.1

Supplementary Table S1C. Estimated marginal means (EMMs) for time to first fixation (TTFF, ms) according to race/ethnicity, emotional expression, and area of interest (AOI).

White Faces

Emotion	Eyes	Mouth	Nose	Whole Face	Non-social
**Contempt**	969.6	2467.1	1242.5	200.1	2826.6
**Happiness**	775.6	1291.4	1518.4	65.1	3361.9
**Fear**	817.1	1227.2	1770.7	61.8	3218.1
**Neutral**	797.0	2718.2	1191.7	65.2	2798.8
**Disgust**	592.2	2286.0	1612.7	186.0	2804.6
**Anger**	237.7	2927.1	2365.9	209.9	2719.8
**Surprise**	691.7	1383.6	1593.7	186.4	3076.8
**Sadness**	611.0	2291.9	1254.9	109.4	3081.0

Black Faces

Emotion	Eyes	Mouth	Nose	Whole Face	Non-social
**Contempt**	795.9	1899.4	2191.0	92.5	1875.5

**Happiness**	793.7	1236.4	1231.3	260.7	2872.4
**Fear**	355.1	1883.7	2008.9	179.0	2105.8
**Neutral**	626.1	2500.1	1395.2	286.2	1392.8
**Disgust**	1020.9	1627.8	1292.3	51.6	2367.0
**Anger**	582.3	3828.5	2288.1	74.8	2323.6
**Surprise**	1295.9	1708.4	1374.7	157.6	1426.0
**Sadness**	685.9	2497.4	1674.3	94.6	2382.9

General note for Supplementary Tables S1A–S1C

Note. Estimated marginal means (EMMs) were derived from linear mixed-effects models fitted by restricted maximum likelihood (REML), including participant as a random intercept. Values are presented as estimated means (ms). AOIs comprised eyes, mouth, nose, whole face, and non-social regions. Means in Table S1A were averaged across stimulus sex, race/ethnicity, and observer sex. Means in Tables S1B and S1C were collapsed across observer sex. Pairwise comparisons were adjusted using Bonferroni correction. These tables correspond to the significant interactions Emotion × AOI, Stimulus Sex × Emotion × AOI, and Race/Ethnicity × Emotion × AOI reported in the main text.

Supplementary Table S2

Number of Fixations (NF)

Supplementary Table S2A. Estimated marginal means (EMMs) for fixation counts (FC) according to observer sex and area of interest (AOI).

AOI	Girls	Boys
**Eyes**	7.559 (0.178)	6.672 (0.145)
**Mouth**	2.025 (0.178)	1.626 (0.145)
**Nose**	2.629 (0.178)	2.255 (0.145)
**Whole Face**	13.957 (0.180)	12.848 (0.147)
**Non-social**	2.541 (0.178)	4.207 (0.145)

Supplementary Table S2B. Estimated marginal means (EMMs) for fixation counts (FC) according to stimulus sex and area of interest (AOI).

AOI	Female Faces	Male Faces
**Eyes**	6.758 (0.155)	7.555 (0.171)
**Mouth**	1.719 (0.155)	1.958 (0.171)
**Nose**	2.475 (0.155)	2.401 (0.171)
**Whole Face**	13.093 (0.155)	13.783 (0.176)
**Non-social**	3.680 (0.155)	2.997 (0.171)

Supplementary Table S2C. Estimated marginal means (EMMs) for fixation counts (FC) according to race/ethnicity and area of interest (AOI).

AOI	White Faces	Black Faces
**Eyes**	7.386 (0.139)	6.783 (0.191)
**Mouth**	1.822 (0.139)	1.831 (0.191)
**Nose**	2.382 (0.139)	2.516 (0.191)
**Whole Face**	13.521 (0.143)	13.256 (0.191)
**Non-social**	2.977 (0.139)	3.862 (0.191)

Supplementary Table S2D. Estimated marginal means (EMMs) for fixation counts (FC) according to emotional expression and area of interest (AOI).

Emotion	Eyes	Mouth	Nose	Whole Face	Non-social
**Contempt**	6.660 (0.302)	1.256 (0.302)	2.273 (0.302)	12.785 (0.302)	3.814 (0.302)
**Happiness**	6.205 (0.330)	2.668 (0.330)	2.785 (0.330)	13.433 (0.330)	3.590 (0.330)
**Fear**	7.628 (0.330)	1.869 (0.330)	2.178 (0.330)	13.665 (0.330)	3.395 (0.330)
**Neutral**	7.785 (0.302)	1.181 (0.302)	2.594 (0.302)	13.788 (0.302)	3.433 (0.302)
**Disgust**	6.995 (0.330)	2.398 (0.330)	2.786 (0.330)	13.819 (0.330)	2.421 (0.330)
**Anger**	8.136 (0.330)	0.910 (0.330)	1.760 (0.330)	12.821 (0.330)	3.870 (0.330)
**Surprise**	6.865 (0.320)	2.830 (0.320)	2.705 (0.320)	14.326 (0.370)	3.292 (0.320)
**Sadness**	6.643 (0.310)	1.443 (0.310)	2.518 (0.310)	12.755 (0.310)	3.281 (0.310)

Note. Estimated marginal means (EMMs) were derived from linear mixed-effects models fitted by restricted maximum likelihood (REML), including participant as a random intercept. Values are presented as estimated fixation counts (FC). Areas of interest (AOIs) comprised eyes, mouth, nose, whole face, and non-social regions. Means in Tables S2A–S2C were averaged across emotion and the remaining factors. Means in Table S2D were averaged across observer sex, stimulus sex, and race/ethnicity.

Total Fixation Duration (TFD)

Supplementary Table S2E. Estimated marginal means (EMMs) for total fixation duration (TFD) according to observer sex, emotion, and area of interest (AOI)

Sex	Emotion	Eyes	Mouth	Nose	Whole Face	Non-social
**Female**	Contempt	2961.93	653.74	950.24	5542.06	922.39
**Female**	Happiness	2881.16	1241.03	1049.34	5879.01	749.23
**Female**	Fear	3230.65	1101.51	947.95	5989.77	735.81
**Female**	Neutral	3358.76	599.53	906.59	5673.75	1038.17
**Female**	Disgust	2754.06	1662.49	949.10	5910.12	571.45
**Female**	Anger	3966.03	348.01	504.28	5469.62	848.14
**Female**	Surprise	3002.21	1441.92	1006.69	5854.42	841.19
**Female**	Sadness	3030.97	734.94	971.72	5531.64	901.72
**Male**	Contempt	2475.33	424.36	639.56	4503.70	1438.98
**Male**	Happiness	1990.09	1075.33	804.28	4601.48	1331.86
**Male**	Fear	2647.42	574.58	469.44	4560.49	1373.65
**Male**	Neutral	2972.70	363.51	666.02	4903.86	964.78
**Male**	Disgust	2299.74	1041.41	630.64	4563.83	828.76
**Male**	Anger	2832.18	302.85	598.48	4495.85	1533.50
**Male**	Surprise	2169.28	909.58	680.39	4425.43	1190.85
**Male**	Sadness	2452.52	589.01	722.32	4481.97	1167.28

Note. Estimated marginal means (EMMs) were derived from linear mixed-effects models fitted by restricted maximum likelihood (REML), with participant included as a random intercept. Values represent total fixation duration (TFD, ms).
